# Poly(A) tail dynamics, non-adenine incorporation and alternative polyadenylation shape the host transcriptome in COVID-19 pathogenesis

**DOI:** 10.1038/s41598-025-21969-5

**Published:** 2025-10-30

**Authors:** Mateusz A. Maździarz, Katarzyna Krawczyk, Ewa Lepiarczyk, Łukasz Paukszto, Karol G. Makowczenko, Beata Moczulska, Piotr Iwanowicz, Piotr Kocbach, Krzysztof Nosek, Jakub Sawicki, Leszek Gromadziński, Marta Majewska

**Affiliations:** 1 Department of Botany and Evolutionary Ecology, Faculty of Biology and Biotechnology, University of Warmia and Mazury in Olsztyn, Olsztyn, 10-721 Poland; 2 Department of Human Physiology and Pathophysiology, School of Medicine, Collegium Medicum, University of Warmia and Mazury in Olsztyn, Olsztyn, 10-082 Poland; 3 Team of Programming of Fertility and Development, Institute of Animal Reproduction and Food Research of Polish Academy of Sciences, Olsztyn, 10-683 Poland; 4 Department of Cardiology and Internal Medicine, School of Medicine, Collegium Medicum, University of Warmia and Mazury in Olsztyn, Olsztyn, 10-082 Poland; 5 Diagnostyka Medical Laboratories, Olsztyn, 10-082 Poland; 6 Department of Family Medicine and Infectious Diseases, School of Medicine, Collegium Medicum, University of Warmia and Mazury in Olsztyn, Olsztyn, 10-082 Poland; 7 Department of Pharmacology and Toxicology, School of Medicine, Collegium Medicum, University of Warmia and Mazury, Olsztyn, 10-082 Poland

**Keywords:** SARS-CoV-2, COVID-19, Blood, Poly(A), Non-A, Computational biology and bioinformatics, Molecular biology, Molecular medicine

## Abstract

The COVID-19 pandemic has had a profound global impact since its emergence in late 2019. Characterized by a wide spectrum of clinical manifestations, COVID-19 has necessitated extensive research into the host-pathogen interactions that drive disease progression. Understanding the molecular mechanisms underlying the host response to SARS-CoV-2 infection is crucial for the development of effective therapeutic interventions and preventative strategies. This study employed a multi-omic approach that combined direct RNA sequencing (DRS) and Illumina cDNA sequencing to investigate whole blood transcriptomic profiles in COVID-19 patients. By leveraging the unique capabilities of Nanopore DRS, which provides long-read sequencing data, we were able to capture not only gene expression levels but also crucial poly(A) tail length fluctuations and non-adenine residue (non-A) modifications. This comprehensive analysis allowed us to identify differentially expressed genes and explore the impact of these poly(A) modifications on function within the context of COVID-19. Findings reveal significant alterations in gene expression patterns, poly(A) tail lengths and non-A modifications in COVID-19 patients compared to healthy controls. Results provide valuable insights into the complex interplay between viral infection, host immune response, and RNA processing, contributing to a deeper understanding of COVID-19 pathogenesis.

**Supplementary Information:**

The online version contains supplementary material available at 10.1038/s41598-025-21969-5.

## Introduction

The first documented case of COVID-19 emerged in Wuhan, China, in December 2019. Since then, the global case burden has surpassed 775 million, with over 7 million reorted deaths (https://covid19.who.int/), which had unprecedented social and economic consequences. Advancements in prophylactic vaccination strategies have significantly mitigated the pandemic’s severity^1^, leading the World Health Organization (WHO) to declare the end of the COVID-19 public health emergency in May 2023 (https://news.un.org/en/story/2023/05/1136367). However, the emergence of novel variants with the potential to trigger surges in cases and mortality remains a concern, especially since the etiological agent responsible for COVID-19 disease, severe acute respiratory syndrome coronavirus 2 (SARS-CoV-2)^2^, unlike other respiratory viruses, does not follow normal seasonal fluctuations, and waves of infection can happen at any time of year^3,4^. Its genome encodes a repertoire of viral proteins, categorized into non-structural proteins crucial for viral replication and pathogenesis and structural proteins essential for virion assembly^5,6^.

Despite the rapid global characterization of clinical symptoms associated with SARS-CoV-2 infection, a comprehensive understanding of the underlying host response and pathogenic mechanisms that govern disease progression toward recovery or fatality remains elusive. Elucidating the molecular foundations of COVID-19 pathogenesis is crucial for developing efficacious preventive and therapeutic strategies, ultimately aiming to reduce mortality and viral transmission. In our previous study, we investigated key genes engaged in SARS-CoV-2 infection using Illumina TruSeq RNA sequencing (RNA-seq) of peripheral blood samples collected from healthy donors and COVID-19 patients^7^. This time, to gain the most extensive insight into the whole blood transcriptomic profiles of the SARS-CoV-2-infected patients, we decided to exploit both RNA-seq short reads and Nanopore long reads. Nanopore sequencing technology enables direct, real-time analysis of long fragments of RNA in fully scalable formats.

The transcripts obtained as a result of native RNA long reads provide a range of valuable information, such as poly(A) tail and (non-A) information^8–10^. Polyadenylation, the process of adding a tail of adenosine nucleotides (poly(A) tail) to the 3’ end of messenger RNA (mRNA), is a critical step in post-transcriptional gene regulation. This modification affects mRNA stability, nuclear export, and translational efficiency. Shorter poly(A) tails are typically associated with reduced translation and increased degradation, whereas longer tails enhance mRNA stability and translation potential. In recent years, studies have also uncovered that poly(A) tails are not composed exclusively of adenosine residues; enzymes such as terminal nucleotidyltransferase 4 A (TENT4A) and terminal nucleotidyltransferase 4B (TENT4B) can incorporate non-adenine nucleotides (cytosine, guanine, or uracil) into the tail, introducing heterogeneity. These non-A residues may serve as regulatory signals by interfering with deadenylation enzymes, thereby influencing RNA fate. In the context of viral infections, including SARS-CoV-2, such dynamic modifications of the RNA tail may profoundly affect the host’s ability to mount an effective antiviral response^11,12^.

This in-depth understanding of molecular changes induced by the virus is of utmost scientific and clinical importance, as it sheds light on the intricate interplay between viral infection, endothelial dysfunction, and immune responses in COVID-19. Such insights can guide the development of targeted therapeutic strategies to tackle the disease effectively.

## Materials and methods

### Patients and sample collection

A total of 20 peripheral blood samples were included in this study: 10 from healthy controls (CTR1–CTR10) and 10 from COVID-19 patients (P1–P10). Among these, 12 samples were subjected to both Illumina short-read RNA sequencing and Nanopore direct RNA sequencing. This group included all 10 control samples and two COVID-19 patient samples (P1 and P4). The remaining COVID-19 samples were sequenced using only one platform: P2, P3, P5, and P6 with Illumina, and P7 through P10 with Nanopore. A full sample-to-platform assignment is provided in Suppl. Fig. S1. The subjects with confirmed cases of COVID-19 were enrolled at the Clinical Department of Communicable Diseases in Ostróda, Poland. Importantly, none of the COVID-19 patients had a known prior SARS-CoV-2 infection before hospitalisation, as determined through clinical records and interviews. All control individuals tested negative and had no medical history suggesting previous exposure or infection. A peripheral arterial oxygen saturation level ≤ 93% was one of the criteria used for hospital admission, according to the institutional protocol. Moreover, patients fulfilled the requisite criteria for a viral diagnosis of SARS-CoV-2, with viral genes confirmed by RT-PCR analysis of nasopharyngeal swabs. The RT-PCR reactions were conducted using the commercially available COVID-19 Real-Time Multiplex RT-PCR Kit (Labsystems Diagnostics OY, Vantaa, Finland). The kit is designed to detect the ORF1ab, N, and E genes of the SARS-CoV-2 genome in a single reaction. The RT-PCR reactions were conducted following the manufacturer’s recommended protocol, and the results were analysed using a QuantStudio™ 5 Real-Time PCR System instrument. The inclusion criteria comprised a positive PCR test for SARS-CoV-2, as well as a clinical diagnosis of COVID-19 requiring hospitalisation. The SARS-CoV-2 infected patients (5 females, 5 males) aged between 54 and 90, revealed a range of pulmonary changes consistent with SARS-CoV-2 infection. The majority of patients demonstrated bilateral inflammatory infiltrates within the lung parenchyma, with varying degrees of severity. The extent of pulmonary involvement ranged from mild (2–3% of lung volume) to severe (up to 90%), with a notable case of advanced bilateral involvement reaching approximately 85% of lung volume. Ground-glass opacities (GGO) and areas of consolidation were commonly observed, often accompanied by fibrotic streaks and, in several instances, pleural effusions of up to 5 cm. One patient exhibited features typical of organising pneumonia, including diffuse alveolar opacities and a “crazy paving” pattern, which involved over 70% of lung tissue. Mediastinal lymphadenopathy was noted in select cases, particularly those with advanced radiologic severity. Importantly, radiologic findings in at least three patients were explicitly described as characteristic of COVID-19 pneumonia. Overall, the spectrum of imaging abnormalities aligns with the known pulmonary manifestations of COVID-19, ranging from mild interstitial involvement to widespread alveolar damage, consolidation, and post-inflammatory fibrosis.

Laboratory findings highlighted systemic inflammation and multi-organ involvement. C-reactive protein (CRP) levels were markedly elevated in several patients, reaching as high as 234 mg/L (normal: 0–5 mg/L), consistent with severe inflammatory response. Aspartate aminotransferase (AST) and alanine aminotransferase (ALT) were also elevated in some cases, peaking at 147 U/L and 116 U/L, respectively, suggesting hepatic involvement. D-dimer levels, a marker of coagulopathy and thrombotic risk, were substantially raised in certain patients, notably one with a value of 548.6 ng/mL, and another with 34.74 ng/mL, significantly exceeding the normal threshold. Leukocyte counts varied, with some patients showing mild leukocytosis (up to 8.9 × 10³/µL) and others within normal ranges. Collectively, these laboratory profiles underscore the systemic and multi-organ burden of COVID-19, correlating with radiologic severity. Elevated inflammatory markers (CRP), liver enzymes (ALT/AST) and coagulopathy (D-dimer) are consistent with known biomarkers of poor prognosis in COVID-19 patients.

The exclusion criteria encompassed patients with neoplasms, autoimmune disease, a pressive or immunodeficient state, and human immunodeficiency virus (HIV) infection. The control group was constituted by volunteers who had tested negative for SARS-CoV-2 infection and showed no signs of respiratory tract infections or lung pathologies, as confirmed by a physician. The control group was constituted in compliance with the following inclusion criteria (verified by a screening questionnaire): absence of a history of travel to high-risk areas, lack of admission to the vaccine, lack of known exposure to a proven or suspected case of SARS-CoV-2 in the previous 14 days, absence of upper or lower respiratory tract infection or any other active illness at the time of blood collection, and lack of past or current history of serious chronic disease such as immune disease. Whole blood samples (3 mL) were collected from all patients (1–15) and placed into Tempus™ Blood RNA Tubes (Applied Biosystems, Waltham, Massachusetts, USA). These samples were stored at −80 °C until analysis time.

### Chest computed tomography

Chest computed tomography (CT) was employed as the primary diagnostic tool for managing patients during the initial stages of the severe acute respiratory syndrome coronavirus 2 (SARS-CoV-2) pandemic. All patients underwent a chest CT scan without intravenous contrast in the supine position (Toshiba Medical System, Aquilion Prime type TSX-303 A/BK; a tube kilovoltage (kV), 120–135 kV, tube current 530–600 mA, 160 layers, 80 rows). The scans were analyzed using the Osirix MD 11.0™ software (Pixmeo Company, Bernex, Suiça) by two radiologists with experience in chest CT, without previous knowledge of the RT-PCR results of the individual patients. Chest CT scans were qualitatively assessed to identify opacity types, specifying their morphology, distribution, and percentage of involvement of the lung parenchyma.

### Total RNA extraction from peripheral blood

The total RNA was isolated from the whole blood of both the experimental and control groups using the Tempus™ Spin RNA Isolation Kit (Applied Biosystems, Waltham, Massachusetts, USA). Before extraction, the Tempus tubes containing the patient’s blood were thawed and transferred into a 50 mL tube. Subsequently, 3 mL of PBS (Ca²⁺/Mg²⁺-free) was added to reach a total volume of 12 mL. The tubes were vigorously vortexed for a minimum of 30 s and subsequently centrifuged at 4 °C at 3000×g for 30 min. Following this, the supernatant was carefully poured off, and the RNA pellet was purified under the manufacturer’s instructions. Finally, total RNA quantity and quality were evaluated utilising an Agilent 2100 Bioanalyzer (Agilent Technologies, USA).

### Nanopore direct RNA sequencing (DRS)

Total RNA isolates were enriched for mRNA using NEBNext^®^ Poly(A) mRNA Magnetic Isolation Module (New England Biolabs) which removed ribosomal RNA (rRNA). Long read libraries were then prepared from 50 ng of poly(A)-tailed mRNA per sample using the Direct RNA Sequencing Kit SQK-RNA002 (Oxford Nanopore Technologies) following the manufacturer’s protocol. SuperScript III Reverse Transcriptase (Thermo Fisher Scientific) was used in the first step of library preparation, which is the synthesis of the complementary strand to the RNA, creating an RNA-cDNA hybrid. Next, sequencing adapters were attached using T4 DNA Ligase (2 M U/ml, New England Biolabs) in combination with NEBNext^®^ Quick Ligation Reaction Buffer. The libraries were quantified with the Qubit dsDNA HS Assay Kit (ThermoFisher) and sequenced on a MinION MK1C sequencing device (ONT) using FLO-MIN 106 Flow Cells R.9.4.1 (ONT). The Flow Cells were prepared for sequencing with the Flow Cell Priming Kit EXP-FLP002 (ONT). Long-read digital MinION signals were first converted from POD5 to FAST5 format using the pod5-file-format program (https://github.com/nanoporetech/pod5-file-format). Next, transcriptomic sequences were basecalled by Guppy v.6.0.0 (https://community.nanoporetech.com/docs/prepare/library_prep_protocols/Guppy-protocol/v/gpb_2003_v1_revax_14dec2018/guppy-software-overview).

### Short-read RNA sequencing

RNA sequencing generating short reads was performed by an external service provider, Macrogen (Amsterdam, The Netherlands), utilizing the Illumina technology (Illumina, San Diego, CA, USA). Briefly, the quality (RIN) and quantity of isolated RNA were assessed using the Tapestation 2200 (Agilent Technologies, Santa Clara, CA, USA). Samples with a RIN value greater than 7.0 were selected for further processing. Sequencing libraries were constructed using the Illumina TruSeq Stranded Total RNA with Ribo-Zero Plus rRNA Depletion kit (Illumina, San Diego, CA, USA), following the manufacturer’s protocol outlined in the TruSeq Stranded mRNA Reference Guide (#1000000092426 v01). Generated libraries were quantified using both qPCR and the KAPA Library Quantification Kit (Roche, Pleasanton, CA, USA). Subsequently, libraries were normalized, pooled in equimolar concentrations, and sequenced on the Illumina NovaSeq 6000 platform in a paired-end configuration (2 × 150 bp). Binary base call (BCL) files were converted into FASTQ format using the Illumina bcl2fastq v.2.19 package (https://github.com/brwnj/bcl2fastq). Following conversion, the sequencing data was directed for further analysis.

### Expression profiling based on nanopore DRS

The FASTQ raw reads were then quality-checked and subjected to mapping steps against a reference *Homo sapiens* genome v.GRCh38. This mapping was performed using minimap2 v.2.26 software with *-ax splice* option (10.1093/bioinformatics/bty191). Gene expression profiles were subsequently estimated using featureCounts v.2.0.6 (10.1093/bioinformatics/btt656), based on a GTF file (GRCh38.p14) from which information about hemoglobin coding genes had been removed. A statistical test based on a negative binomial model and shrink, implemented in DESeq2 v.1.42.0^13^ was additionally employed for analysis. The statistical significance of differentially expressed genes (DEGs) was determined using the following parameters: adjusted P-value < 0.05 and |log2FoldChange (log2FC)| > 1.

### Expression profiling based on cDNA illumina sequencing

Raw reads were trimmed using Trimmomatic v.0.39 (https://github.com/usadellab/Trimmomatic)^14^ with the following parameters: *crop: 140*,* leading: 20*,* trailing: 20*,* minlen: 140*,* avgqual: 20*. Next, STAR v.2.7.11b (https://github.com/alexdobin/STAR)^15^ was used to map FASTQ files against *H. sapiens* reference genome v.GRCh38 utilizing ENCODE standard parameters. Gene count information was obtained using featureCounts with the reference GTF file (without hemoglobin genes). Then, similar to DRS, DESeq2 was used to assess the significance of differential gene expression. The fluctuation of gene expression with adjusted P-value < 0.05 and |log2FC| > 1 was considered statistically significant. The Pearson correlation was plotted between the log2FC values from cDNA Illumina sequencing and DRS for molecules that showed statistical significance.

### Differential adenylation

The FASTQ files were remapped into *H. sapiens* transcriptome (GRCh38.p14). Tail information for each transcript was then extracted using the nanopolish v.0.14.1 tool (https://github.com/jts/nanopolish). Subsequently, a statistical method based on the Wilcoxon test was applied by the nanotail v.0.1.0 package (https://github.com/smaegol/nanotail) to determine the differences in tail length between molecules transcribed in both conditions. Only reads tagged by nanopolish as ‘pass’ or ‘suffclip’ were considered in the following analyses. mRNA tails shorter than 10 bp and transcripts with fewer than 10 counts were excluded from the analysis. Poly(A) tails of genes with an adjusted P-value less than 0.05 were considered statistically significant. Statistical significance of overall tail length difference between COVID-19 and control patients was assessed using the Wilcoxon test.

### Non-adenine residue analysis

Previously generated nanopolish outcomes, sequencing summary generated by the Guppy basecaller, and FAST5 files were used to identify non-adenine (non-A) sites in the poly(A) tail. This identification was performed by the ninetails v.1.0.0 software (https://github.com/MystPi/ninetails)^10^.

### Alternative polyadenylation sites detection

The BAM files generated from mapping long reads to a reference genome were utilized to identify and analyze variations in alternative polyadenylation sites (APA). The LAPA program^16^ was used to predict statistically significant APA sites, which considered the following parameters adjusted P-value < 0.05 and |Δ usage| > 0.3.

### Functional annotations

All statistically significant molecules were then scanned for enrichment analysis in Gene Ontology (GO) annotations^17,18^ using the g: profiler v.0.2.2 R package^19^. For the essential genes, biological processes (BP), cellular components (CC) and molecular functions (MF) terms were assigned as ontological annotations. Enrichment analysis was subsequently employed to identify GO terms regulated by significant molecules, using an adjusted P-value < 0.05 cut-off.

### Visualization

The visualizations were generated using the R environment and the following packages: ggplot2 v.3.5.1^20^, ComplexHeatmap v.2.18.0^21^, and ggvenn v.0.1.10 (https://github.com/yanlinlin82/ggvenn). Furthermore, visualizations offered by the previously employed software were leveraged.

## Results

### Chest computed tomography

**Fig. 1:**
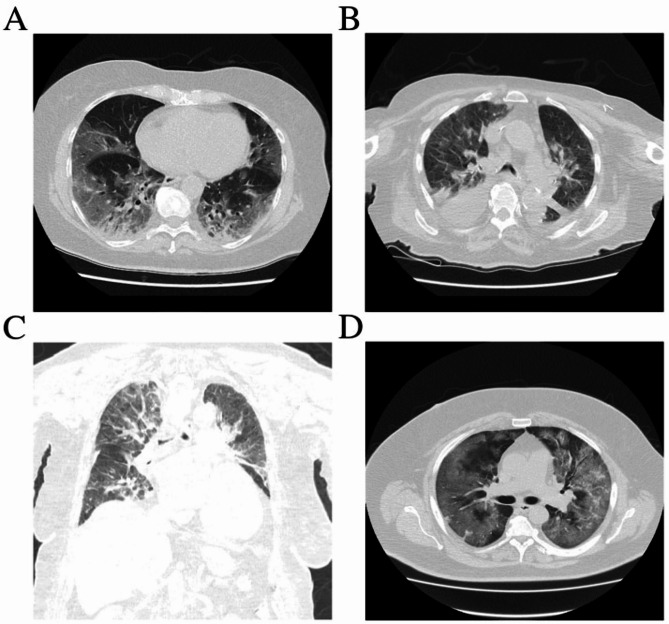
Noncontrast axial and coronal chest CT images showing inflammatory lung changes in hospitalized COVID-19 patients. (**A**) Extensive ground-glass opacities with consolidation, primarily in the lower lobes; ~50% of the lung parenchyma affected. (**B**) Bilateral ground-glass opacities with marked interlobular septal thickening, subsegmental atelectasis or consolidation, bilateral pleural effusions (5 cm right, 2 cm left), and mediastinal lymphadenopathy (16 × 11 mm); ~90% parenchymal involvement. (**C**, **D**) Confluent ground-glass opacities and dense consolidations involving all lobes, most prominent in the left upper lobe; ~70–75% right and 75–80% left lung parenchyma affected.

The most frequently observed CT findings demonstrated a strong correlation with a COVID-19 diagnosis. Representative chest CT scans demonstrated inflammatory changes of varying severity among COVID-19 patients (Fig. 1). In a 67-year-old woman on day 7 of illness (Fig. 1A), imaging revealed extensive ground-glass opacities with consolidation, primarily in the lower lobes, affecting approximately 50% of the lung parenchyma. In a 90-year-old woman on day 2 of illness (Fig. 1B), bilateral ground-glass opacities with marked interlobular septal thickening were observed, consistent with severe pulmonary involvement or pulmonary oedema. Subsegmental atelectasis or consolidation was present in the lower lobes. Pleural effusions measured up to 5 cm on the right and 2 cm on the left, and mediastinal lymphadenopathy was noted, with the largest node measuring 16 × 11 mm. Total lung involvement was estimated at 90%. In a 63-year-old man on day 7 of illness (Fig. 1C, D), CT images showed marked hypoventilation, along with extensive, confluent ground-glass opacities and consolidations involving all lobes. Patchy dense consolidations were most prominent in the upper lobe of the left lung. The extent of parenchymal involvement was estimated at 70–75% in the right lung and 75–80% in the left lung. Peripheral distribution of the lesions, ground-glass opacities, and bronchovascular thickening in the lesions were found to have the highest value in diagnosing COVID-19 patients (Fig. 1).

### Comparative transcriptome analysis of COVID-19 patients reveals distinct gene expression signatures

**Fig. 2:**
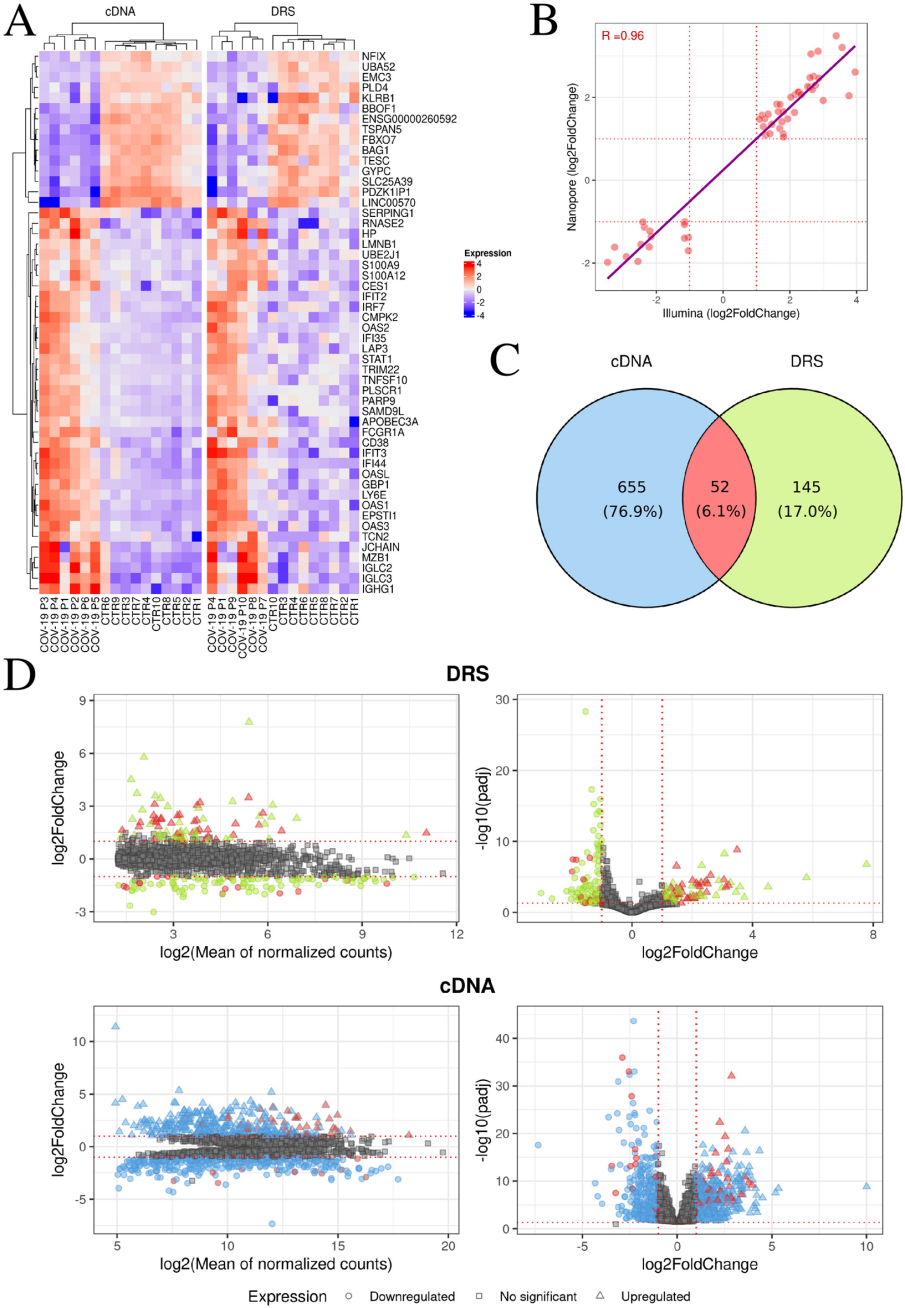
Gene expression profiling of controls and COVID-19 patients. (**A**) The heatmap displays the normalised expression of 52 common genes for DRS and cDNA sequencing. Red colour indicates values greater than 0, while blue indicates values less than 0. (**B**) The scatter plot illustrates the relationship between log2FC values for the 52 DEGs identified in both sequencing methods. The x-axis represents log2FC values for cDNA Illumina sequencing, while the y-axis depicts log2FC values for DRS Nanopore sequencing. The red line highlights the Pearson correlation. The R value is displayed in the upper left corner. (**C**) The Venn diagram presents DEGs in cDNA Illumina (blue), DEGs in DRS Nanopore (green), and common DEGs (red). (**D**) DEGs characteristic of COVID-19 patients. The top panels correspond to DRS Nanopore, while the bottom panels represent cDNA Illumina sequencing. All DEGs are colored according to the Venn diagram. On the left, MA plots show the relationship between log2FC and log2 (Mean of normalised counts), while on the right, Volcano plots show the relationship between -log10(padj) and log2FC.

Direct RNA sequencing (DRS) provided information on the expression of 16,510 genes, of which 197 were identified as differentially expressed genes (DEGs). Among these molecules, 112 were downregulated and 85 were upregulated. The log2FC ranged from − 7.78 to 4.23 for all molecules (Figs. 2A–D; Supplemental Table 1). Subsequently, a GO (Gene Ontology) analysis of all significant molecules was conducted, which were significantly involved in immune response (GO:0006955), response to virus (GO:0009615), and defense response (GO:0006952) (Supplemental Table 2). In the next stage of bioinformatics, cDNA expression generated by Illumina was analyzed. Sequencing using this method provided information on 36,689 genes. A total of 707 molecules were classified as DEGs, including 362 downregulated genes and 345 upregulated genes. The log2FC ranged from − 11.41 to 9.36 for all genes (Figs. 2A–D; Supplemental Table 3). Subsequently, a GO analysis of all significant molecules was conducted, which were significantly involved in immune response (GO:0006955), response to virus (GO:0009615), and defense response (GO:0006952) (Supplemental Table 4). The next step involved plotting the Pearson correlation between the log2FC of common genes. A total of 52 DEGs common to the cDNA and DRS methods were classified. Among the common genes, nuclear factor IX (*NFIX)*, leucine aminopeptidase 3 (*LAP3)*, immunoglobulin lambda constant 3 (*IGLC3)*, sterile alpha motif domain containing 9 like (*SAMD9L)*, and interferon induced protein with tetratricopeptide repeats 3 (*IFIT3)* were identified (Figs. 2A,C). The correlation coefficient between their expression log2FC values was 0.96, with a correlation P-value of < 2.2e-16 and a 95% confidence interval of 0.9393051 to 0.9797827. It was found that similar expression signatures were indicated by the results of the correlation coefficients for common genes (Fig. 2B).

### Dissecting the mRNA landscape of COVID-19: A role for poly(A) tail dynamics

**Fig. 3:**
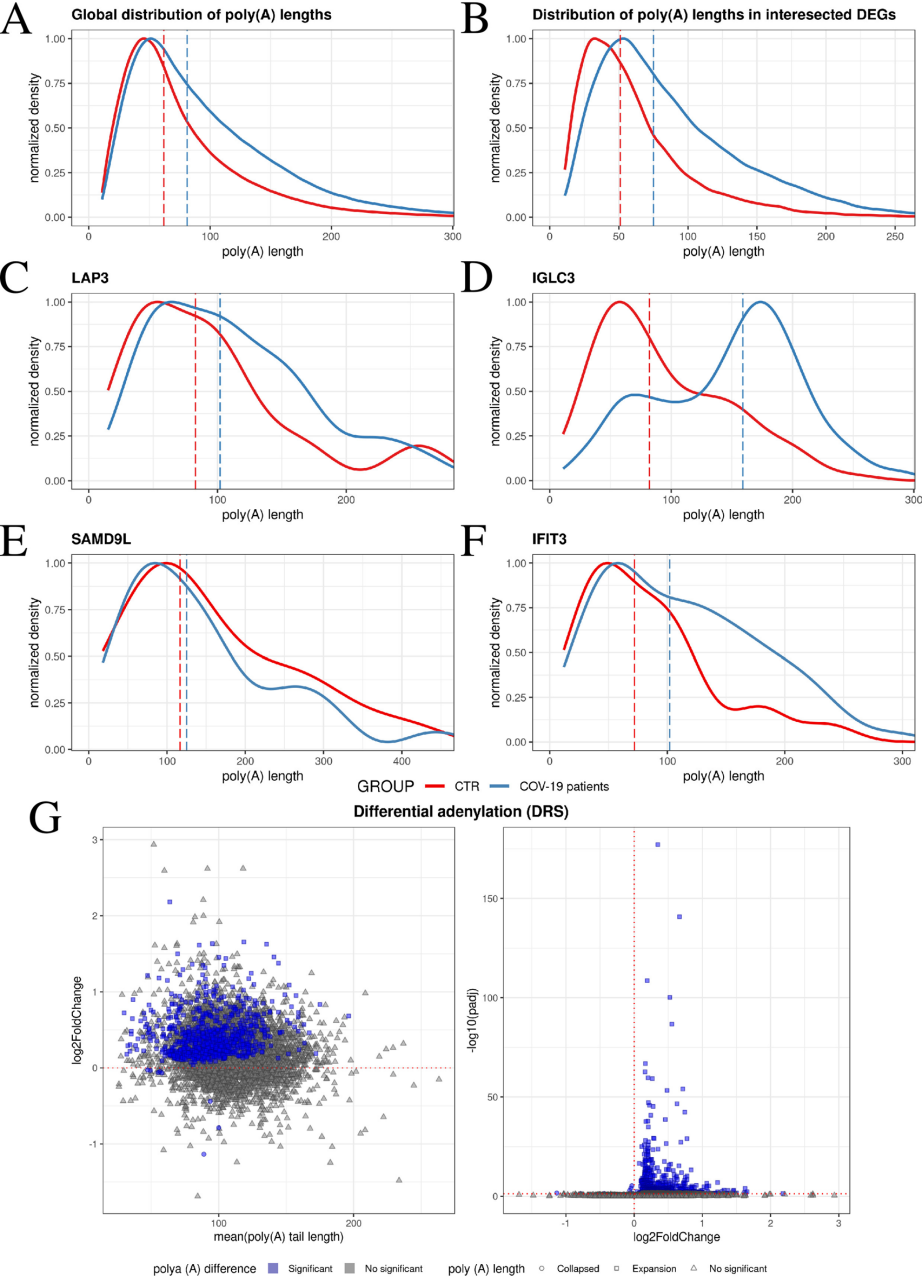
Poly(A) tail length variation across the genes. (**A**–**F**) The density plots depict the normalized transcript density on the y-axis and the length of poly(A) tails on the x-axis. Panel (**A**) shows the global distribution of poly(A) tails, panel (**B**) shows the poly(A) distribution of intersected DEGs, while panels (**C**) to (**F**) present the poly(A) distribution for specific genes: *LAP3*, *IGLC3*, *SAMD9L*, and *IFIT3*, respectively. (**G**). Illustrates genes with distinct poly(A) characteristics. The left panel displays a modified MA plot, depicting the relationship between log2FC (based on poly(A) tail length) and the mean poly(A) tail length. Conversely, the right panel presents a Volcano plot, showcasing the relationship between log2FC (based on poly(A) tail length) and -log10(padj). Significant genes are highlighted in blue, with shortened tails represented by circles and lengthened tails denoted by squares.

A total of 2,029,252 poly(A) tails were analyzed. A statistically significant difference in the global distribution of poly(A) tail lengths was found between COVID-19 and control patients (P-value < 2.2e-16). 6,524 transcripts with poly(A) tails were identified (Fig. 3A; Supplemental Table S5). Lengthening of poly(A) tails was observed in as many as 879 genes in COVID-19 patients, compared to only 8 in the control group (Fig. 3G; Supplemental Table S5). Subsequently, a GO analysis of all significant transcripts was conducted, which were significantly involved in immune response (GO:0006955), response to virus (GO:0009615), and defense response (GO:0006952) (Supplemental Table 6). Furthermore, differences in poly(A) tail lengths in differentially expressed genes (DEGs) common to both methods were examined. A significant result was obtained, indicating a lengthening of poly(A) tails in DEGs of COVID-19 patients (P-value < 2.2e-16) (Fig. 3B). Additionally, differences in poly(A) tail lengths were examined for individual genes: *LAP3* (P-value = 0.046) (Fig. 3C), *IGLC3* (P-value < 2.2e-16) (Fig. 3D), *SAMD9L* (P-value = 0.7385) (Fig. 3E), and *IFIT3* (P-value = 0.026) (Fig. 3F).

**Fig. 4:**
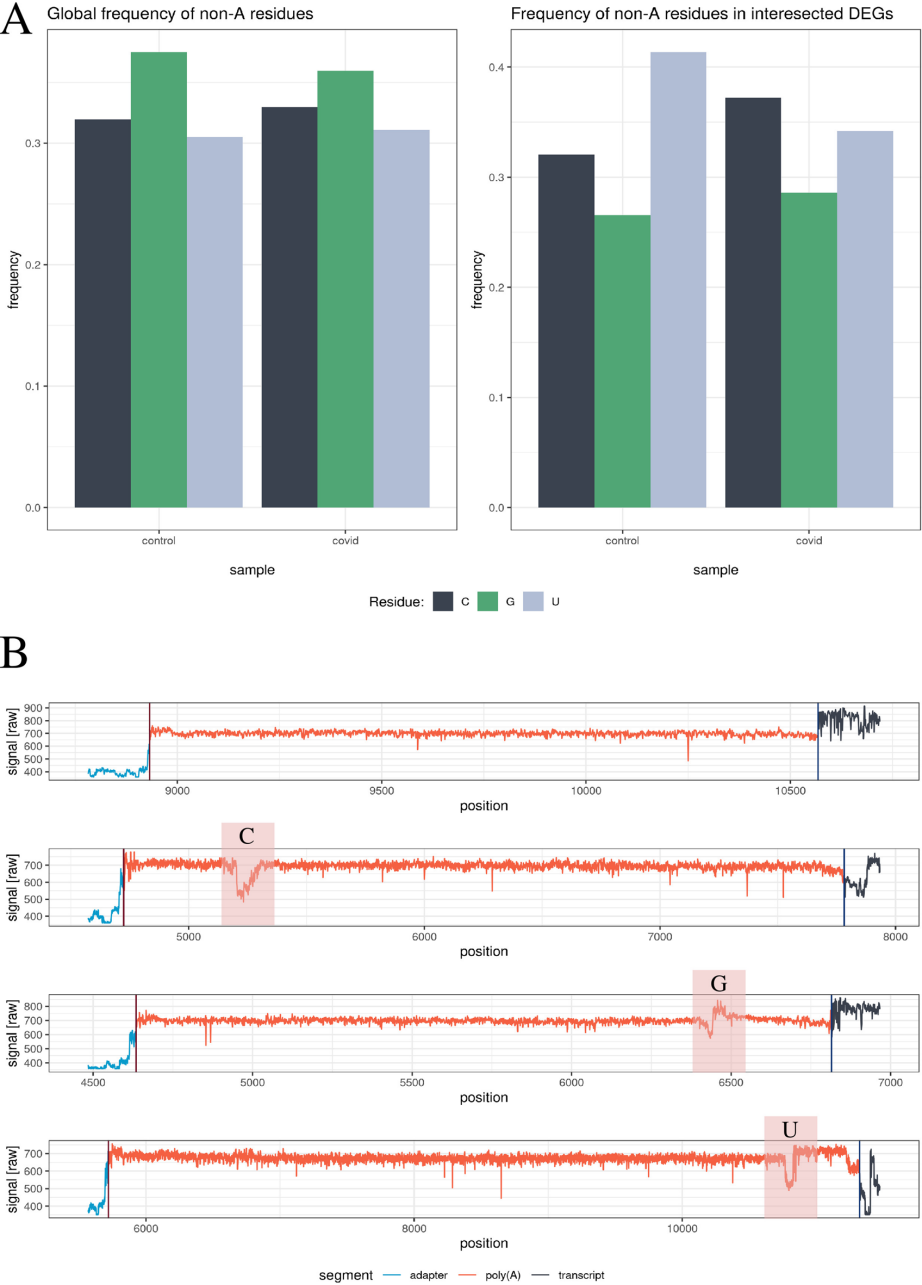
Characterization of non-A residues in COVID-19 and control patients samples. (**A**) non-A occurrence patterns. The left panel visualizes the overall frequency of non-A residues in control and COVID-19 patient samples. The right panel depicts the distribution of non-A residues within differentially expressed genes (DEGs) shared between both groups. (**B**) Poly(A) tail illustration. The blue signal represents the adapter, the red signal indicates the poly(A) tail and the black signal corresponds to the transcript. The first panel showcases a canonical poly(A) tail, while subsequent panels demonstrate poly(A) tails with cytosine, guanine, or uracil substitutions, respectively.

In addition to adenine, other nucleotides such as guanine, uracil, and cytosine were identified within poly(A) tails. A global analysis of the frequency of non-adenine (non-A) residues was conducted, revealing that guanine (22,129 for COVID-19 patients and 15,447 for control patients) was found more frequently poly(A) tails than cytosine (20,300 for COVID-19 patients and 13,165 for control patients) or uracil (19,141 for COVID-19 patients and 12,576 for control patients) (Fig. 4A; Supplemental Table 7). A shift in this trend was observed in DEGs common to both methods. Cytosine (911) was determined to be the most frequent non-A residue in COVID-19 patients, whereas uracil (556) was found to be the most frequent in the control group. Guanine (700 for COVID-19 patients and 357 for control patients) was identified as the least frequent non-A residue in both groups (Figs. 4A, B; Supplemental Table 8).

### Alternative polyadenylation and its impact on COVID-19

**Fig. 5:**
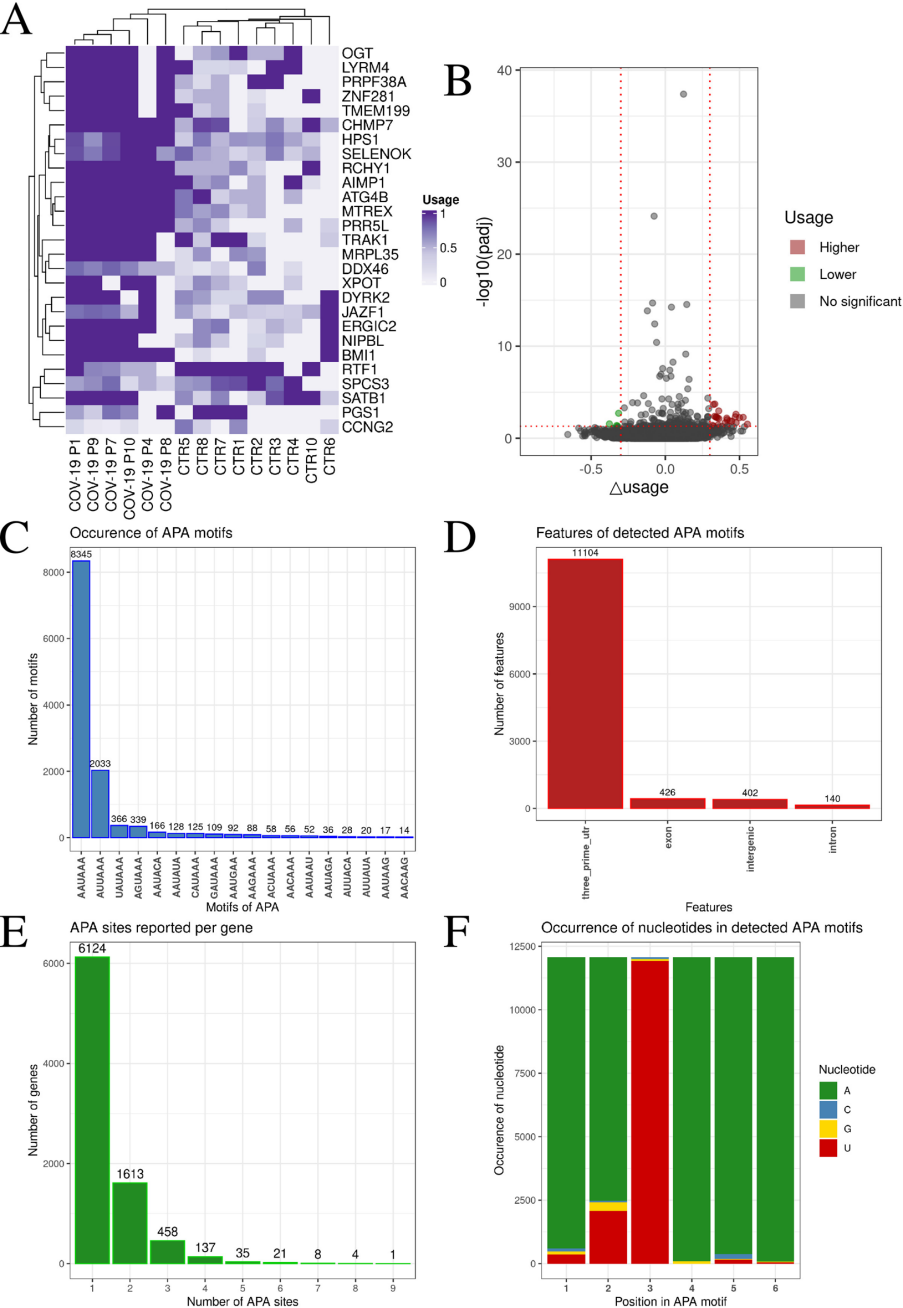
Identification of APA site patterns and associated features. (**A**) Heatmap visualizes use of APA sites in genes. Darker purple indicates a value closer to 1. Gene names are listed on the right, and patient names are listed at the bottom of the heatmap. (**B**) Volcano plot shows the relationship between -log10(padj) and Δusage. Gray dots represent statistically insignificant APA sites, green dots represent statistically significant APA sites with Δusage < −0.3, and red dots represent statistically significant APA sites with Δusage > 0.3. (**C**) Barplots show the frequency of each motif. The x-axis shows the APA motifs and the y-axis shows their count. (**D**) Barplots show the location of APA sites and their frequency. The x-axis shows the specific location, and the y-axis shows the number of APA sites at that location. (**E**) Barplots show the number of APA sites per gene. The x-axis shows the number of APA sites, and the y-axis shows the frequency of that number of APA sites in the groups. (**F**) Barplots show nucleotide frequencies at each position of the detected APA sites. The x-axis shows the position number, and the y-axis the nucleotide frequency. Adenine is represented by green, cytosine by blue, guanine by yellow, and uracil by red.

The most frequent alternative polyadenylation (APA) motifs identified were AAUAAA (8,345) and AUUAAA (2 033) (Figs. 5C, F; Supplemental Table 9). APA motifs were found to be most abundant in the three prime untranslated regions (3’UTR) – 11,104 times, in exons − 426 times, in intergenic regions − 402 times, and in introns − 140 times (Fig. 5D). APA sites were observed to occur in various proportions: 1 site was identified in 6 124 genes, 2 sites in 1 613 genes, and 3 sites in 458 genes (Fig. 5E). Analysis of APA site use differences in genes identified 27 significant genes with APA sites, of which 23 were found to have a Δusage value greater than 0.3 and 4 were found to have a Δusage value less than − 0.3 (Figs. 5A, B; Supplemental Table 10). Genes exhibiting significantly different APA site usage towards COVID-19 patients included BMI1 proto-oncogene, polycomb ring finger (*BMI1)*, JAZF zinc finger 1 (*JAZF1)*, NIPBL cohesin loading factor (*NIPBL)*, and DEAD-box helicase 46 (*DDX46)*, while in control patients, RTF1 homolog, Paf1/RNA polymerase II complex component (*RTF1)*, phosphatidylglycerophosphate synthase 1 (*PGS1)*, signal peptidase complex subunit 3 (*SPCS3)*, and cyclin G2 (*CCNG2)* were identified (Fig. 5A). The GO analysis of genes with significant APA revealed the involvement of six significant processes: (GO:1902494), protein-containing complex (GO:0032991), intracellular protein-containing complex (GO:0140535), nucleoplasm (GO:0005654), nuclear lumen (GO:0031981), intracellular membrane-bounded organelle (GO:0043231) (Supplemental Table 11).

### Summary of multi-omic analyses in COVID-19 patients samples

**Fig. 6:**
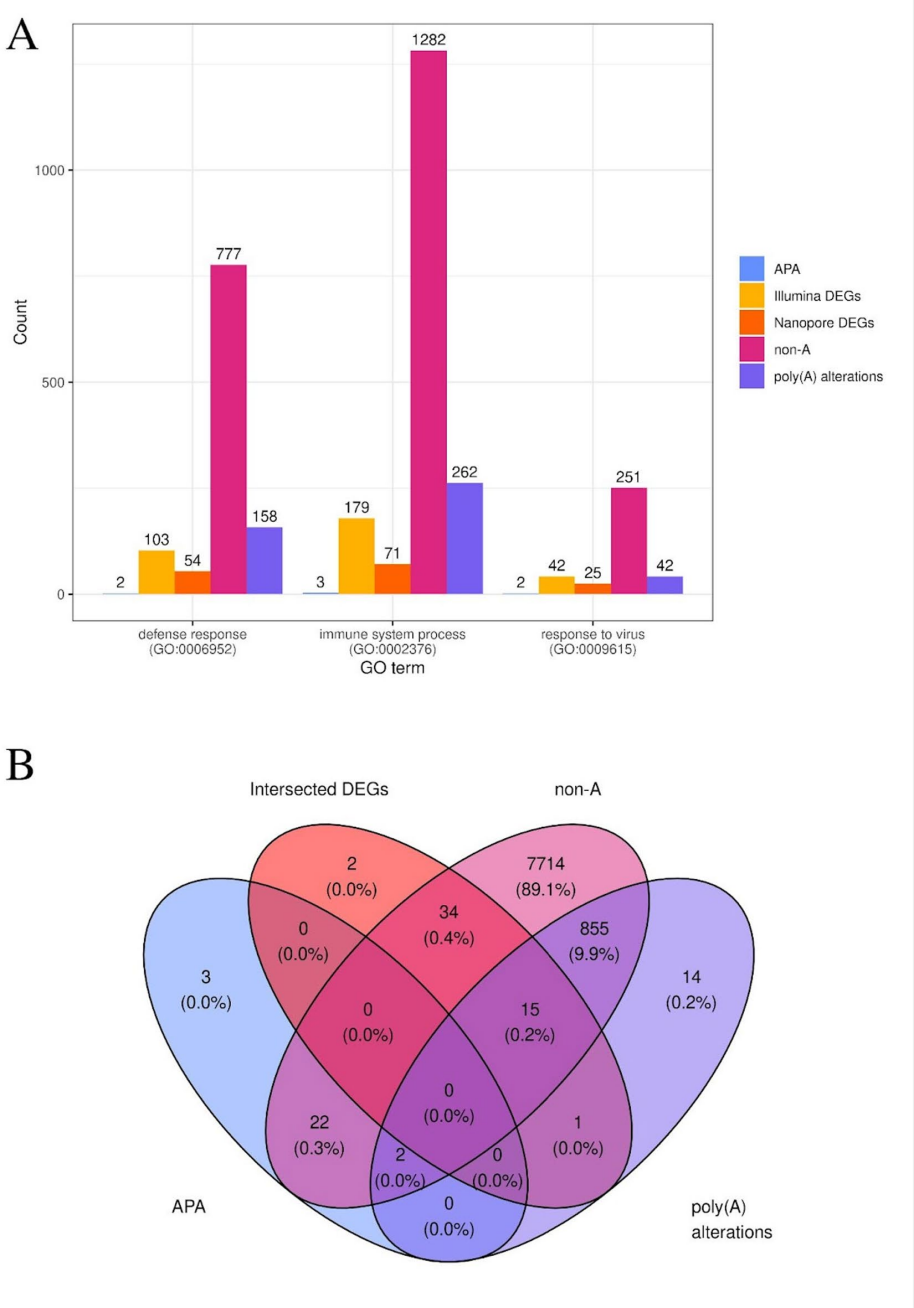
Summary of multi-omic analysis results. (**A**) Barplot depicting Gene Ontology (GO) analysis of all molecules involved in three key processes: defense response, immune system process, and response to virus. Each bar plot is colored according to the legend on the right. (**B**) Venn diagram illustrating the overlaps between differentially expressed genes (DEGs) identified by both sequencing methods (red), poly(A) alterations (purple), alternative polyadenylation (APA) events (blue), and non-adenylated transcripts (pink).

All multi-omic analysis results were functionally evaluated to elucidate the significance of various molecular elements within three pivotal processes: host defense response, immune system processes, and antiviral response. Within the host defense response process, a total of 802 genes were observed to be involved, comprising 2 APA events, 103 Illumina DEGs, 54 Nanopore DEGs, 777 non-A, and 158 poly(A) alterations. Conversely, the antiviral response process encompassed 258 genes, including 2 APA events, 42 Illumina DEGs, 25 Nanopore DEGs, 251 non-A, and 42 poly(A) alterations. Furthermore, in the immune system processes, 1331 genes were identified, consisting of 3 APA events, 179 Illumina DEGs, 71 Nanopore DEGs, 1282 non-A, and 262 poly(A) alterations (Fig. 6A, Supplemental Table 12). It was observed that 855 genes exhibiting significantly altered poly(A) tail lengths also possessed non-A in their poly(A) tails. This indicates that a substantial 96% of genes with significant differences in poly(A) tails were also characterized by non-A. Crucially, no common elements were detected across all four investigated categories. This absence of overlap may suggest a complex and multi-layered molecular and immunological response to SARS-CoV-2 infection (Fig. 6B).

## Discussion

Differential expression analysis, which involves counting the number of reads per gene in an RNA sequence, has become the primary method for identifying systematic changes across experimental conditions^22^. Studies have shown that combining two sequencing methods can provide additional information through gene expression analysis. Furthermore, it has been shown that these methods could be complementary^23^. The present investigation identified 52 DEGs common to both cDNA and DRS methods (Fig. 2A,C). GO analysis of these DEGs categorized them into immune response, response to virus, and defense response terms (Supplemental Table 4). Among the common genes, 2’−5’-oligoadenylate synthetase (*OAS1*), apolipoprotein B mRNA editing enzyme catalytic subunit 3 A (*APOBEC3A*) and interferon induced protein 44 (*IFI44*) were identified (Fig. 2A). The present study revealed that the SARS-CoV-2 infection was followed by the upregulation of *OAS1* which was earlier recognized as a strong antiviral factor against this virus. It has been found that the inactivation of OAS1’s catalytic activity resulted in the loss of its antiviral function by activating RNase L, which degrades both cellular and viral RNA^24^. Investigations have demonstrated that another gene upregulated in COVID-19 patients, namely *APOBEC3A*, is a critical factor in the inhibition of coronaviruses, by restricting the RNA virus replication^25^. A similar result most probably is exerted by an overexpression of *IFI44* in COVID-19 patients. *IFI44* belongs to interferon (IFN)-stimulated genes and has been previously found to control respiratory syncytial virus (RSV) infection by exerting antiviral and antiproliferative properties^26^.

The primary objective of the present study, however, was not to focus on the differential expression of genes detected in the SARS-CoV-2 infected patients, as we have already described them in our previous study^7^, but to leverage the advantages of direct RNA long-read sequencing. Poly(A) tails were found to be dynamic elements of transcripts, rather than static units simply marking the 3’ end of mRNA, which significantly influence the post-transcriptional regulation of the fragile balance of mRNA survival-degradation^27^. The analysis of these structures may provide novel insights into the post-transcriptional regulation of gene expression in the contexts of development, differentiation, and various disease states^28^. In our study, lengthening of poly(A) tails was observed in as many as 879 genes in COVID-19 patients, compared to only 8 in the control group (Fig. 3G; Supplemental Table 5). The GO analysis of all transcripts with significant poly(A) length changes revealed their significant contribution to terms such as immune response, response to virus and defense response again (Supplemental Table 6). This intriguing observation raises a question: why do post-transcriptionally modified transcripts exhibit increased length? As the dynamic part of the poly(A) tail is also longer in these transcripts possibly this elongation may enhance the recruitment of ribosomes and thus facilitate translation initiation. Investigations conducted on *Xenopus laevis* oocytes demonstrated no discernible disparity in the translation of mRNAs with poly(A) tails of 32 or 150 nucleotides in length^29,30^. Moreover, it was found that transcripts with poly(A) tails shorter than 16 nucleotides were not translated. Furthermore, the frequency of molecules possessing fewer than 30 adenines at the 3’ end of the poly(A) tail was significantly lower within the cellular environment. During current research, we identified a change of poly(A) tails median length between the COVID-19 (81 nt) and control patients (62 nt). Conversely, extended poly(A) tails may safeguard genes from rapid enzymatic degradation^31,32^. In the present study, DRS method detected significant poly(A) tail elongation in DEGs identified in COVID-19 patients for instance in the *IFIT3*, *IGLC3*,* LAP3* or *SAMD9L*. IFIT3 restricts viral spread by binding to viral RNA and inhibiting translation^33^. Moreover, IFIT3 amplifies the interferon response by stabilizing the IFIT1 and IFIT2 protein complexes, further enhancing their antiviral effects and upregulating the expression of IFN-stimulated genes (ISGs), including the previously described OAS1^34–37^. Type I and type II interferons are crucial components of the innate immune response to viral infections, including SARS-CoV-2^38^. A significant upregulation of the ISGs has been detected during COVID-19 disease. Overexpression of *IFIT3* has been detected in uninfected or asymptomatic females who were repeatedly exposed to their symptomatic COVID-19 male partners^39^. Given its potential role in antiviral immunity, the *IFIT3* offers a promising avenue for understanding the mechanisms underlying protection from SARS-CoV-2 infection. Elucidating these mechanisms may have significant implications for developing new therapeutic strategies. Nonstructural protein 16 (NSP16) is essential to the SARS-CoV-2 replication cycle because it is essential to coronavirus’ immune evasion^40^. NSP16 is a 2′-O-methyltransferase (2′-O-MTase) that forms part of the replication-transcription complex^41^. Inhibition of NSP16 enhances SARS-CoV-2 susceptibility to IFN-I-induced antiviral effectors, such as IFIT1 and IFIT3^42^. Patients with severe COVID-19 displayed increased B cell activation and upregulation of IGLC3, a marker of antibody processing. These findings suggest a robust antibody response to enhance the host protection and enhanced interferon signaling in these individuals^43^. Single-cell RNA sequencing revealed *IGLC3* among the other 15 differentially expressed genes between patients who survived COVID-19^44^. Accurate identification of prognostic factors in critical COVID-19 patients can aid in risk assessment and guide tailored therapeutic interventions. SARS-CoV-2 infection alters the host gene expression profile, leading to the upregulation of interferon-stimulated genes, including *LAP3*. The interferon IFN-stimulated ISG15 had the largest increase in serum of COVID-19 patients, followed by several other IFN-induced proteins, such as LAP3^45^ . Similarly, *SAMD9L* was among the differentially regulated interferon-stimulated genes in mild and severe disease cohorts, suggesting that it may play a critical role in SARS-CoV-2 pathogenesis^46^. Previous studies demonstrated that the SAMD9L pathway acts as a crucial host defense mechanism, which poxviruses actively suppress to establish infection^47^. This pathway was identified among the interferon-stimulated genes exhibiting significantly reduced expression in patients with severe COVID-19 compared to those with mild cases. The identification of *SAMD9L* as a downregulated gene in severe COVID-19 highlights its potential role as a critical host restriction factor that SARS-CoV-2 must overcome to establish infection^46^. Moreover, in people with severe COVID-19 the infection reveals a diminished antiviral response marked by the downregulation of antiviral genes such as *OAS1*, *SAMD9L* and *IFIT2*, and suppression of antiviral immune response pathways^48^.

Shortening the 3’UTR through alternative polyadenylation (APA) may be a key mechanism contributing to COVID-19 pathogenesis. APA-mediated reduction of 3’UTR length can increase gene expression by evading miRNA-mediated silencing during SARS-CoV-2 infection^49^. Moreover, global 3′UTR shortening affects protein abundance, and the impact of the 3′UTR on protein production may depend on the gene. However, the APA of the genes confers different functions and needs further investigation. It was observed that the expression of 3′ processing factors was down-regulated when cells were infected by vesicular stomatitis virus, which might be one of the reasons underlying genome-wide APA when cells were infected with viruses. It has been found that the expression level of 3′ processing factors is also altered in COVID-19 patients. SARS-CoV-2 proteins can bind to APA factors affecting the gene expression level of APA factors to regulate this process^49^. Additionally, alternative polyadenylation has been suggested to impair antigen presentation by MHC molecules in infected cells. Disrupting alternative polyadenylation and splicing could further enhance the ability of SARS-CoV-2 to evade the host immune response^50^.

Within the dataset analyzed in this study, 4 out of 27 genes (14,8%) showed the 3′UTR shortening mechanism *via* APA (Δusage < −0.3), providing specific examples of potential escape from miRNA regulation in COVID-19 patients: *CCNG2*, *PGS1*, *RTF1* and SPCS3 (Fig. 5A, B; Supplemental Table 10). The first of these genes encodes cyclin G2 and has documented binding sites for miR-590-3p (three sites in the 3’UTR)^51^, miR-1290^52^, miR-1246^53^, miR-17-5p^54^ and miR-378a-5p^55^. Shortening the 3’UTR of this gene may lead to the elimination of critical miRNA binding sites, allowing escape from repression and dysregulation of cell cycle control. *PGS1* encodes an enzyme involved in the biosynthesis of cardiolipin, which in turn plays an important role in the proper functioning of the IV complex of the respiratory chain. Despite the lack of available literature data on the post-transcriptional regulation of *PGS1* by miRNA, the occurrence of such a process under certain physiological or pathological conditions cannot be excluded. In this context, shortening its 3’UTR may affect mitochondrial homeostasis, which is particularly relevant in the pathophysiology of COVID-19 The RTF1 protein is a component of the Paf1/RNA polymerase II transcription complex and is a confirmed target of at least 21 human miRNAs evidenced within the mirTarBase database^56^ (including CLIP-Seq method). As a regulator of transcription elongation, its escape from miRNA control may have broad implications for the global regulation of gene expression in COVID-19. The last of the aforementioned genes, *SPCS3*, encodes a component of the signal peptidase complex in the endoplasmic reticulum (ER). Its 3’UTR has been confirmed to contain binding sites for 60 miRNA molecules in mirTarBase (including CLIP-Seq, microarray and pSILAC methods). Dysregulation of this gene may affect protein processing and the response to ER stress. Additionally, the miRDB database^57^, based on predictive algorithms, indicates the presence of 215 miRNAs targeting the 3’UTR of the *CCNG2* gene in the human, 48 miRNAs for *PGS1*’s 3’UTR, 174 miRNAs for *RTF1* and 199 miRNAs for *SPCS3*. These examples demonstrate a mechanism whereby shortening the 3’UTR by shifting it towards proximal polyadenylation sites can eliminate miRNA regulatory elements, leading to increased expression of genes critical for the immune and metabolic response in COVID-19.

The poly(A) tail of mRNA has been conventionally considered a homogenous stretch of adenosine nucleotides, devoid of significant information content beyond its length. However, the non-canonical poly(A) polymerases, TENT4A and TENT4B have been identified as enzymes capable of incorporating non-A nucleotides, such as guanine, uracil, and cytosine, into the poly(A) tail^11^. While the function of this mechanism remains unclear, it is hypothesized that the presence of these non-adenine nucleotides (non-A mutations) may impede the activity of deadenylase enzymes, slowing the rate of poly(A) tail shortening and increasing the mRNA stability^11,27^. However, Poly(A)-binding protein can stimulate the removal of adenine residues from the poly(A) tail, a process known as deadenylation. This contrasts with the expectation that stable, highly translated mRNAs would possess longer poly(A) tails^31^. It has to be mentioned that the present study revealed also the non-A residues in both samples from COVID-19 patients and control group, and SARS-CoV-2-infected patients exhibited increased cytosine content and decreased guanine content in non-A residues (Fig. 4A, B; Supplemental Table 7). Recent studies have highlighted that TENT4A and TENT4B can introduce non-canonical residues such as C and U into poly(A) tails of host mRNAs, producing “mixed tailing” patterns that influence mRNA stability and translation efficiency. This non-A tailing has been shown to confer partial protection against rapid deadenylation by canonical deadenylases such as the CCR4-NOT complex^11,58^. Specifically, C and U additions can disrupt the processivity of deadenylases and thereby prolong the half-life of selected mRNA transcripts.

The increased incorporation of non-adenine residues (especially cytosine and uracil) into poly(A) tails in COVID-19 patients may suggest functional consequences. In the context of viral infection, such as with SARS-CoV-2, it is plausible that the observed increase in C and U residues within host mRNA poly(A) tails reflects a host-driven regulatory response, potentially aimed at stabilizing antiviral transcripts or modulating immune-related gene expression. Alternatively, it may reflect viral subversion of host RNA processing machinery to enhance the stability of viral or proviral host mRNAs. Notably, recent findings have implicated TENT4A/B in the stabilization of mRNAs encoding components of the antiviral response, such as interferon-stimulated genes (ISGs)^59^.

Taken together, our findings reveal that SARS-CoV-2 infection induces profound remodeling of the host RNA landscape, extending beyond classical gene expression changes to include poly(A) tail elongation, non-adenine incorporation, and 3′UTR architecture alterations. These multilayered post-transcriptional modifications likely act in a coordinated manner to enhance mRNA stability and translational efficiency of immune effector transcripts such as *IFIT3*, *SAMD9L*, and *LAP3*, thereby amplifying antiviral responses and supporting interferon signaling. Importantly, the observed enrichment of cytosine residues and APA-mediated 3′UTR shortening in key immune-related genes may represent an adaptive mechanism to evade miRNA-mediated repression during viral stress. By leveraging long-read sequencing, we captured these subtle but highly coordinated transcriptomic shifts with single-molecule resolution. Future research incorporating longitudinal sampling, mechanistic studies, and integrative analysis of RNA processing dynamics will be critical for understanding how SARS-CoV-2 shapes the host post-transcriptional regulatory network to influence disease trajectory.

### Study limitations

While this study provides novel insights into RNA tailing dynamics during SARS-CoV-2 infection, several limitations should be acknowledged. The sample size, although in line with comparable exploratory transcriptomic studies under pandemic constraints, may limit the statistical power of some analyses, particularly those involving APA and non-adenine tail composition. In addition, sampling was limited to a single time-point per subject, which constrains the temporal interpretation of transcriptomic shifts. Despite these factors, robust differences were consistently observed between groups, underscoring the biological relevance of the findings. Future studies involving larger, multi-centre cohorts with longitudinal designs and richer clinical metadata will be valuable in further validating and extending these observations. Although all COVID-19 patients in our cohort had no documented prior infection and healthy controls were rigorously screened to exclude past SARS-CoV-2 exposure, we acknowledge that undetected asymptomatic infections, particularly among controls, cannot be fully excluded. Such prior exposures may influence transcriptional outcomes through mechanisms such as immune imprinting or trained immunity, potentially affecting baseline gene expression and poly(A) tail dynamics. Trained immunity, characterized by long-term functional reprogramming of innate immune cells following infection or vaccination, has been shown to modulate antiviral responses and transcriptomic states independent of active infection^60,61^. While our selection criteria minimized this confounding factor, future studies incorporating serological screening and longitudinal sampling would provide a more definitive understanding of how immunological memory impacts RNA processing and host-virus interaction profiles.

## Conclusions

To the best of our knowledge, this is the first study to decipher in such a deep extent both Nanopore long reads and RNA-seq datasets to investigate the whole blood transcriptomic profiles of the SARS-CoV-2 infected patients by providing comprehensive insights into the epitranscriptome features and post-transcriptional modifications. The identification of DEGs such as *OAS1*, *APOBEC3A*, and *IFI44*, as well as genes associated with immune responses, highlights the robust activation of antiviral pathways during COVID-19. The investigation revealed extensive poly(A) tail lengthening in COVID-19 patients, particularly among immune-related transcripts, suggesting an adaptive mechanism to enhance transcript stability and translation efficiency. Exploring further host–SARS-CoV-2 interactions at a deep molecular level may be a fascinating focus for research for future therapeutic treatment.

## Supplementary Information

Below is the link to the electronic supplementary material.

Supplementary Material 1

Supplementary Material 2

## Data Availability

The authors confirm that all data generated and analyzed during this study are either included in this published article or available from the corresponding authors upon reasonable request. The data underlying this article are available in the European Nucleotide Archive repository, under accession numbers PRJEB84380, PRJEB74103 (https://www.ebi.ac.uk/ena/browser/view/PRJEB84380, https://www.ebi.ac.uk/ena/browser/view/PRJEB74103).

## Abbreviations

APA: Alternative polyadenylation
APOBEC3A: Apolipoprotein B mRNA editing enzyme catalytic subunit 3A
BAM: Binary alignment map
BCL: Base call file
BMI1: BMI1 proto-oncogene, polycomb ring finger
BP: Biological process
CC: Cellular component
CCNG2: Cyclin G2
COVID-19: Coronavirus disease 2019
CT: Computed tomography
DDX46: DEAD-box helicase 46
DEG: Differentially expressed GENE
DRS: Direct RNA sequencing
ENCODE: Encyclopedia of DNA elements
ER: Endoplasmic reticulum
FAST5: Fast format 5
FASTQ: Fast format Q
GGO: Ground-glass opacities
GO: Gene ontology
GTF: Gene transfer format
HIV: Human immunodeficiency virus
IFI44: Interferon induced protein 44
IFIT3: Interferon induced protein with tetratricopeptide repeats 3
IGLC3: Immunoglobulin lambda constant 3
ISGs: IFN-stimulated genes
JAZF1: JAZF zinc finger 1
LAP3: Leucine aminopeptidase 3
log2FoldChange: log2FC
MF: Molecular function
NFIX: Nuclear factor IX
NIPBL: NIPBL cohesin loading factor
NSP16: Nonstructural protein 16
OAS1: 2’-5’-oligoadenylate synthetase
PBS: Phosphate-buffered saline
PCR: Polymerase chain reaction
PGS1: Phosphatidylglycerophosphate synthase 1
RBPs: RNA-binding proteins
RIN: RNA integrity number
RNA-seq: RNA sequencing
RT-PCR: Real-time polymerase chain reaction
RTF1: RTF1 homolog, Paf1/RNA polymerase II complex component
SAMD9L: Sterile alpha motif domain containing 9 Like
SARS-CoV-2: Severe acute respiratory syndrome coronavirus 2
SPCS3: Signal peptidase complex subunit 3
STAR: Spliced transcripts alignment to a reference
TENT4A: Terminal nucleotidyltransferase 4A
TENT4B: Terminal nucleotidyltransferase 4B
UTR: Untranslated region
WHO: World Health Organization
bp: Base pair
cDNA: Complementary DNA
mRNA: Messenger RNA
non-A: Non-adenine residue
qPCR: Quantitative PCR
rRNA: Ribosomal RNA

## References

[1] Rahmani, W. et al. Attenuation of SARS-CoV-2 infection by Losartan in human kidney organoids. *iScience* **25**, 103818 (2022). 35106453 10.1016/j.isci.2022.103818 PMC8795780

[2] Zhu, N. et al. A novel coronavirus from patients with pneumonia in China, 2019. *N Engl. J. Med.* **382**, 727–733 (2020). 31978945 10.1056/NEJMoa2001017 PMC7092803

[3] Silk, B. J. et al. COVID-19 surveillance after expiration of the public health emergency declaration ― united States, May 11, 2023. *MMWR Morb Mortal. Wkly. Rep.* **72**, 523–528 (2023). 37167154 10.15585/mmwr.mm7219e1 PMC10208372

[4] Zhang, H. et al. Recent developments in the immunopathology of COVID-19.. *Allergy: European Journal of Allergy and Clinical Immunology* **78**, 369–388. 10.1111/all.15593 (2023). 36420736 10.1111/all.15593 PMC10108124

[5] Astuti, I. & Ysrafil Severe acute respiratory syndrome coronavirus 2 (SARS-CoV-2): an overview of viral structure and host response. *Diabetes Metabolic Syndrome: Clin. Res. Reviews*. **14**, 407–412 (2020). 10.1016/j.dsx.2020.04.020 PMC7165108 32335367

[6] Uddin, M. et al. SARS-CoV-2/COVID-19: viral Genomics, Epidemiology, Vaccines, and therapeutic interventions. *Viruses* **12**, 526 (2020). 32397688 10.3390/v12050526 PMC7290442

[7] Majewska, M. et al. SARS-CoV-2 disrupts host gene networks: unveiling key hub genes as potential therapeutic targets for COVID-19 management. *Comput. Biol. Med.* **183**, 109343 (2024). 39500239 10.1016/j.compbiomed.2024.109343

[8] Wang, Y., Zhao, Y., Bollas, A., Wang, Y. & Au, K. F. Nanopore sequencing technology, bioinformatics and applications. *Nat. Biotechnol.* **39**, 1348–1365 (2021). 34750572 10.1038/s41587-021-01108-x PMC8988251

[9] MacKenzie, M. & Argyropoulos, C. An introduction to nanopore sequencing: Past, Present, and future considerations. *Micromachines (Basel)*. **14**, 459 (2023). 36838159 10.3390/mi14020459 PMC9966803

[10] Gumińska, N. et al. Direct profiling of non-adenosines in poly(A) tails of endogenous and therapeutic mRNAs with Ninetails. *Nat. Com.* **16**(1), 1–16 (2025). 10.1038/s41467-025-57787-6 PMC11920217 40102414

[11] Lim, J. et al. Mixed tailing by TENT4A and TENT4B shields mRNA from rapid deadenylation. *Sci. (1979)*. **361**, 701–704 (2018). 10.1126/science.aam5794 30026317

[12] Liudkovska, V. & Dziembowski, A. Functions and mechanisms of RNA tailing by metazoan terminal nucleotidyltransferases. *Wiley Interdiscip Rev. RNA*. **12**, e1622 (2021). 33145994 10.1002/wrna.1622 PMC7988573

[13] Love, M. I., Huber, W. & Anders, S. Moderated Estimation of fold change and dispersion for RNA-seq data with DESeq2. *Genome Biol.* **15**, 1–21 (2014). 10.1186/s13059-014-0550-8 PMC4302049 25516281

[14] Bolger, A. M., Lohse, M. & Usadel, B. Trimmomatic: a flexible trimmer for illumina sequence data. *Bioinformatics* **30**, 2114–2120 (2014). 24695404 10.1093/bioinformatics/btu170 PMC4103590

[15] Dobin, A. et al. STAR: ultrafast universal RNA-seq aligner. *Bioinformatics* **29**, 15 (2012). 23104886 10.1093/bioinformatics/bts635 PMC3530905

[16] Çelik, M. H. & Mortazavi, A. Analysis of alternative polyadenylation from long-read or short-read RNA-seq with LAPA. *bioRxiv* 11.08.515683 (2022) (2022). 10.1101/2022.11.08.515683

[17] Ashburner, M. et al. Gene ontology: tool for the unification of biology. The gene ontology consortium. *Nat. Genet.* **25**, 25–29 (2000). 10802651 10.1038/75556 PMC3037419

[18] Carbon, S. et al. Expansion of the gene ontology knowledgebase and resources: the gene ontology consortium. *Nucleic Acids Res.* **45**, D331–D338 (2017). 27899567 10.1093/nar/gkw1108 PMC5210579

[19] Kolberg, L. et al. G:Profiler-interoperable web service for functional enrichment analysis and gene identifier mapping (2023 update). *Nucleic Acids Res.* **51**, W207–W212 (2023). 37144459 10.1093/nar/gkad347 PMC10320099

[20] Villanueva, R. A. M. & Chen, Z. J. ggplot2: elegant graphics for data analysis. *Meas. (Mahwah N J)*. **17**, 160–167 (2019). 2nd ed.

[21] Gu, Z., Eils, R. & Schlesner, M. Complex heatmaps reveal patterns and correlations in multidimensional genomic data. *Bioinformatics* **32**, 2847–2849 (2016). 27207943 10.1093/bioinformatics/btw313

[22] Love, M. I., Anders, S., Kim, V. & Huber, W. RNA-Seq workflow: gene-level exploratory analysis and differential expression. *F1000Research* **4**, 1070 (2016). 10.12688/f1000research.7035.1 PMC4670015 26674615

[23] Maździarz, M. et al. Epitranscriptome insights into Riccia fluitans L. (Marchantiophyta) aquatic transition using nanopore direct RNA sequencing. *BMC Plant. Biol.* **24**, 1–16 (2024). 38745128 10.1186/s12870-024-05114-4 PMC11094948

[24] Danziger, O., Patel, R. S., DeGrace, E. J., Rosen, M. R. & Rosenberg, B. R. Inducible CRISPR activation screen for Interferon-Stimulated genes identifies OAS1 as a SARS-CoV-2 restriction factor. *PLoS Pathogens* **18** (2022). 10.1371/journal.ppat.1010464 PMC9041830 35421191

[25] Milewska, A. et al. APOBEC3-mediated restriction of RNA virus replication. *Sci. Rep.* **8**, 1–12 (2018). 29654310 10.1038/s41598-018-24448-2 PMC5899082

[26] Busse, D. C. et al. Interferon-Induced protein 44 and Interferon-Induced protein 44-Like restrict replication of respiratory syncytial virus. *J. Virol.* **94**, e00297–e00220 (2020). 32611756 10.1128/JVI.00297-20 PMC7459546

[27] Liu, Y., Nie, H., Zhang, Y., Lu, F. & Wang, J. Comprehensive analysis of mRNA poly(A) tail reveals complex and conserved regulation. *BioRxiv* **2021.08.29.458068**10.1101/2021.08.29.458068 (2021).

[28] Jalkanen, A. L., Coleman, S. J. & Wilusz, J. Determinants and implications of mRNA poly(A) tail size–does this protein make my tail look big? *Semin Cell. Dev. Biol.* **34**, 24–32 (2014). 24910447 10.1016/j.semcdb.2014.05.018 PMC4163081

[29] NUDEL, U. et al. Globin mRNA species containing Poly(A) segments of different lengths. *Eur. J. Biochem.* **64**, 115–121 (1976). 776610 10.1111/j.1432-1033.1976.tb10279.x

[30] Mercer, J. F. B. & Wake, S. A. An analysis of the rate of Metallothionein mRNA POLY(A)-shortening using RNA blot hybridization. *Nucleic Acids Res.* **13**, 7929–7943 (1985). 2866488 10.1093/nar/13.22.7929 PMC322101

[31] Passmore, L. A. & Coller, J. Roles of mRNA poly(A) tails in regulation of eukaryotic gene expression. *Nat. Rev. Mol. Cell Biol.* **23**(2), 93–106 (2021). 34594027 10.1038/s41580-021-00417-y PMC7614307

[32] Alles, J., Legnini, I., Pacelli, M. & Rajewsky, N. Rapid nuclear deadenylation of mammalian messenger RNA. *iScience* **26**, 105878 (2023). 36691625 10.1016/j.isci.2022.105878 PMC9860345

[33] Hsu, J. C. C., Laurent-Rolle, M. & Cresswell, P. Translational regulation of viral RNA in the type I interferon response. *Curr. Res. Virol. Sci.* **2**, 100012 (2021).

[34] Chai, B. et al. Murine ifit3 restricts the replication of rabies virus both in vitro and in vivo. *J. Gen. Virol.* **102**, 001619 (2021). 10.1099/jgv.0.001619 34269675

[35] Chikhalya, A. et al. Human IFIT3 protein induces interferon signaling and inhibits adenovirus immediate early gene expression. *mBio* 12, (2021). 10.1128/mBio.02829-21 PMC8561380 34724821

[36] Imaizumi, T. et al. IFIT proteins are involved in CXCL10 expression in human glomerular endothelial cells treated with a Toll-Like receptor 3 agonist. *Kidney Blood Press. Res.* **46**, 74–83 (2021). 33326977 10.1159/000511915

[37] Xu, S. et al. IFIT3 is increased in serum from patients with chronic hepatitis B virus (HBV) infection and promotes the Anti-HBV effect of interferon alpha via JAK-STAT2 in vitro. *Microbiol Spectr* **10**, (2022). 10.1128/spectrum.01557-22 PMC9769971 36314949

[38] Watson, A. et al. Dynamics of IFN-β responses during respiratory viral infection insights for therapeutic strategies. *Am. J. Respir Crit. Care Med.* **201**, 83–94 (2020). 31461630 10.1164/rccm.201901-0214OC

[39] de Castro, M. V. et al. Potential protective role of interferon-induced protein with tetratricopeptide repeats 3 (IFIT3) in COVID-19. *Front. Cell. Infect. Microbiol.* **14**, 1464581 (2024). 39664492 10.3389/fcimb.2024.1464581 PMC11631949

[40] Daffis, S. et al. methylation of the viral mRNA cap evades host restriction by IFIT family members. *Nature* **468**(7322), 452–456 (2010). 21085181 10.1038/nature09489 PMC3058805

[41] Vithani, N. et al. SARS-CoV-2 Nsp16 activation mechanism and a cryptic pocket with pan-coronavirus antiviral potential. *Biophys. J.* **120**, 2880–2889 (2021). 33794150 10.1016/j.bpj.2021.03.024 PMC8007187

[42] Schindewolf, C. et al. SARS-CoV-2 uses nonstructural protein 16 to evade restriction by IFIT1 and IFIT3. *J Virol* **97**, (2023). 10.1128/jvi.01532-22 PMC9973020 36722972

[43] Garduno, A. et al. Parallel dysregulated immune response in severe forms of COVID-19 and bacterial sepsis via Single-Cell transcriptome sequencing. *Biomedicines* **11**, 778 (2023). 36979757 10.3390/biomedicines11030778 PMC10045101

[44] Amrute, J. M. et al. Cell specific peripheral immune responses predict survival in critical COVID-19 patients. *Nat. Com.* **13**(1), 1–11 (2022). 10.1038/s41467-022-28505-3 PMC8847593 35169146

[45] Babačić, H. et al. omprehensive proteomics and meta-analysis of COVID-19 host response. *Nat. Com.* **14**(1), 1–18 (2023). 10.1038/s41467-023-41159-z PMC10516886 37739942

[46] Sandi, J. D. et al. Upper Airway Epithelial Tissue Transcriptome Analysis Reveals Immune Signatures Associated with COVID-19 Severity in Ghanaians. *J. Immunol. Res.* **2024**, 6668017 (2024). 38375062 10.1155/2024/6668017 PMC10876312

[47] Meng, X. et al. A paralogous pair of mammalian host restriction factors form a critical host barrier against poxvirus infection. *PLoS Pathog*. **14**, e1006884 (2018). 29447249 10.1371/journal.ppat.1006884 PMC5831749

[48] Goodnow, C. C. COVID-19, varying genetic resistance to viral disease and immune tolerance checkpoints. *Immunol. Cell. Biol.* **99**, 177–191 (2021). 33113212 10.1111/imcb.12419 PMC7894315

[49] Jia, X. et al. The role of alternative polyadenylation in the antiviral innate immune response. *Nat. Com.* **8**(1), 1–12 (2017). 10.1038/ncomms14605 PMC5333124 28233779

[50] An, S. et al. Genome-Wide profiling reveals alternative polyadenylation of innate Immune-Related mRNA in patients with COVID-19. *Front. Immunol.* **12**, 756288 (2021). 34777369 10.3389/fimmu.2021.756288 PMC8578971

[51] Salem, M., Shan, Y., Bernaudo, S. & Peng, C. miR-590-3p targets Cyclin G2 and FOXO3 to promote ovarian cancer cell Proliferation, Invasion, and spheroid formation. *Int. J. Mol. Sci. 2019*. **20**, 1810 (2019). 10.3390/ijms20081810 PMC6515004 31013711

[52] Qin, W. J., Wang, W. P., Wang, X. B., Zhang, X. T. & Du, J. D. MiR-1290 targets CCNG2 to promote the metastasis of oral squamous cell carcinoma. *Eur. Rev. Med. Pharmacol. Sci.* **23**, 10332–10342 (2019). 31841213 10.26355/eurrev_201912_19671

[53] Hasegawa, S. et al. MicroRNA-1246 expression associated with CCNG2-mediated chemoresistance and stemness in pancreatic cancer. *Br. J. Cancer*. **111**, 1572–1580 (2014). 25117811 10.1038/bjc.2014.454 PMC4200094

[54] Huang, Q. et al. miR-17-5p drives G2/M-phase accumulation by directly targeting CCNG2 and is related to recurrence of head and neck squamous cell carcinoma. *BMC Cancer* **21**, (2021). 10.1186/s12885-021-08812-6 PMC8487119 34598688

[55] Nadeem, U., Ye, G., Salem, M. & Peng, C. MicroRNA-378a-5p targets Cyclin G2 to inhibit fusion and differentiation in bewo cells. *Biol Reprod* **91**, (2014). 10.1095/biolreprod.114.119065 25122062

[56] Cui, S. et al. MiRTarBase 2025: Updates to the collection of experimentally validated microRNA-target interactions.. *Nucleic Acids Res.* **53**, D147–D156 (2025). 39578692 10.1093/nar/gkae1072 PMC11701613

[57] Chen, Y. & Wang, X. MiRDB: an online database for prediction of functional MicroRNA targets. *Nucleic Acids Res.* **48**, D127–D131 (2020). 31504780 10.1093/nar/gkz757 PMC6943051

[58] Warkocki, Z., Liudkovska, V., Gewartowska, O., Mroczek, S. & Dziembowski, A. Terminal nucleotidyl transferases (TENTs) in mammalian RNA metabolism. *Philosophical Trans. Royal Soc. B: Biol. Sciences* **373**, (2018). 10.1098/rstb.2018.0162 PMC6232586 30397099

[59] Wen, X., Irshad, A. & Jin, H. The battle for survival: the role of RNA Non-Canonical Tails in the Virus–Host interaction. *Metabolites 2023*. **13**, 1009 (2023). 10.3390/metabo13091009 PMC10537345 37755289

[60] Netea, M. G. et al. The role of trained immunity in COVID-19: lessons for the next pandemic. *Cell. Host Microbe*. **31**, 890–901 (2023). 37321172 10.1016/j.chom.2023.05.004 PMC10265767

[61] Netea, M. G. et al. Defining trained immunity and its role in health and disease. *Nat. Rev. Immunol.* **20**(6), 375–388 (2020). 32132681 10.1038/s41577-020-0285-6 PMC7186935

