# Supplementary figures and images for: Poly(A) tail dynamics, non-adenine incorporation and alternative polyadenylation shape the host transcriptome in COVID-19 pathogenesis
Source: Sci Rep. 2025 Oct 30;15:37986. doi: 10.1038/s41598-025-21969-5 (PMC12575734; doi:10.1038/s41598-025-21969-5)

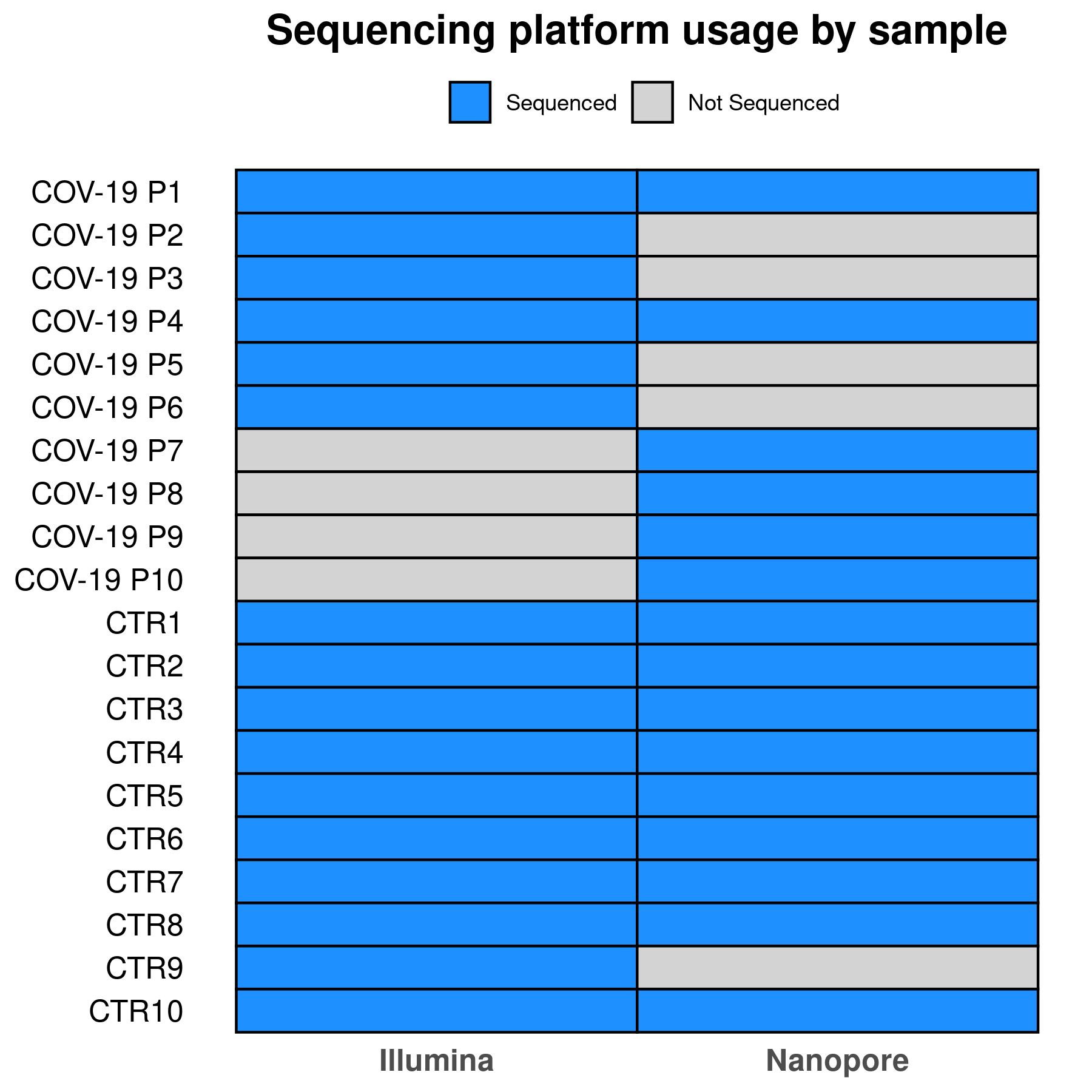

Supplement: Supplementary file 2 — Supplementary Material 2 [file 41598_2025_21969_MOESM2_ESM.png]
